# Comparison of nasal interfaces and pressures during critical time-points in NICU: observation data from over 10 years

**DOI:** 10.1038/s41372-026-02642-9

**Published:** 2026-03-30

**Authors:** Noah H. Hillman, Howard L. Williams, Samrudhi R. Joshi, Lana L. Herrmann, Colleen C. Claassen

**Affiliations:** 1 Division of Neonatology SSM Heath Cardinal Glennon Children’s Hospital, Saint Louis, MO USA; 2 Saint Louis University School of Medicine Department of Pediatrics, Saint Louis, MO USA

**Keywords:** Respiration, Acute inflammation

## Abstract

**Objective:**

The pressure amount and fluctuations delivered to the nasopharynx by bubble CPAP (BCPAP) is affected by the nasal interface, but the clinical effects of transmitted pressure fluctuations from BCPAP are unknown.

**Study design:**

Retrospective study of 2432 infants < 33 weeks GA had respiratory variables electronically extracted for: 1) delivery room, 2) initial 72 h, 3) after extubation, and 4) 31 weeks PMA. BCPAP nasal interfaces (RAM® cannula and Babi.Plus^TM^) for different bottle pressures (5/6 of 7/8 cmH_2_O) were compared.

**Results:**

RAM5/6 had more CPAP failures than RAM7/8 or Babi5/6. Babi5/6 had less CPAP failure in first 72 h in NICU, but RAM7/8 had less in DR. Less infants failed CPAP at extubation in Babi5/6 due to decreased apnea events.

**Conclusion:**

At similar transmitted nasopharyngeal pressures, Babi.Plus had less CPAP failure and apnea than RAM in NICU, whereas the ease of application of RAM may help in delivery room.

## Introduction

Continuous positive airway pressure (CPAP) is recommended for spontaneous breathing preterm infants to decrease rates of bronchopulmonary dysplasia (BPD) [1, 2]. CPAP can help recruit functional residual capacity (FRC) in the delivery room and stabilize breathing patterns in the extremely preterm, so inadequate pressure transmission can increase CPAP failure rates [3, 4]. CPAP success in the delivery room increases with gestational age and may be due to both improvements in respiratory drive and lung compliance [2]. Effective delivery of CPAP to the airways of preterm infants, however, has its challenges clinically and is more nuanced than previously thought. Understanding the role of the delivery of CPAP, with different modalities and airway interfaces, is crucial for improving long-term respiratory health in extremely preterm infants.

Distending pressure in the lungs is essential for proper lung branching and alveolarization in utero, and CPAP can increase alveolarization in premature monkeys and preterm infants [5–7]. In newborn rodent models, CPAP alone can increase airway reactivity but protects against hyperoxia when the two are combined [8]. Underwater bubble CPAP (BCPAP) devices generate a fluctuating pressure wave as the bubble is formed then released [9]. In preterm sheep, the pressure fluctuation with BCPAP can improve oxygenation and ventilation compared to stable CPAP [10]. High-amplitude bubble CPAP (Seattle-CPAP) was designed to further increase these pressure oscillations and can maintain similar blood gas values to mechanical ventilation in paralyzed, saline-lavaged rabbits [11]. Across multiple different institutions, the introduction of BCPAP improved stabilization at birth and decreased delivery room intubations [12, 13], decreased the rates of severe BPD and death [14, 15], and improved cognitive scores at 20 months corrected age [16]. Thus, BCPAP may have physiologic benefits that lead to improved clinical outcomes over static pressure CPAP modes.

Along with variability in the bubble CPAP bottles, there are different nasal interfaces used to deliver BCPAP, and particular interfaces have been shown to attenuate the pressure levels and fluctuations, impacting clinical benefit [17] short binasal prongs (SBPs), such as prongs manufactured by Babi.Plus^TM^, Hudson, and Fisher Paykel, generate the pressure in the nasopharynx, whereas the RAM® cannula generates the CPAP in the cannula circuit connection cap and variable pressure is transmitted down the narrow cannula tubing to the nasopharynx [18, 19]. The RAM cannula use with a BCPAP bottle (RAM CPAP) may act more like a hybrid with high flow nasal cannula (additional information in discussion), and some experts may not consider it BCPAP. We previously demonstrated that increasing the bottle pressure settings with the RAM® cannula, to account for loss of transmitted pressures, decreased CPAP failure rate in delivery room and NICU in the first 72 h [4, 20]. There is a linear relationship, due to resistance in the tubing of the RAM cannula, between pressure set at the bottle and pressure measured at the nares. The choice of CPAP interface by neonatal intensive care units is driven by various factors beyond just pressure delivery, such as user familiarity, bias from past negative experiences, nursing agreeability, costs, and side effects of the interface on the patient’s nasal septum, nostril shape, and head shape.

Our NICUs have made changes in both set CPAP pressures and nasal interfaces over the last ten years. Based on research from our group, our units have moved to using the Babi.Plus (SBPs) as first line, and RAM cannula as second line choice, if nasal septal breakdown occurs despite nasal care plan. We now utilize electronically extracted physiologic data to compare the two interfaces, Babi.Plus and RAM cannula, to evaluate the effects of pressure fluctuations on multiple time periods throughout the infant’s course in the delivery room and NICU.

## Methods

We performed a retrospective study of infants born at <33 weeks of gestation and greater >500 grams between January 1, 2013 and December 31, 2023, either delivered at our women’s hospital SSM Health St. Mary’s Hospital or transferred to our level 4 NICU at SSM Health Cardinal Glennon Children’s Hospital. The cohort was identified using a Microsoft SQL query of the electric medical record (EPIC). Excluded infants included those that were provided comfort care only or had a congenital condition that precluded CPAP use (choanal atresia, diaphragmatic hernia, etc.). The RAM® cannula (Neotech, Valencia, CA) was used off-label as the nasal interface for BCPAP (RAM CPAP) on all infants at birth beginning prior to the study in 2012, and BCPAP bottle pressures gradually increased starting in 2014 and continued until 2019 [4, 20]. Babi.Plus^TM^ nasal interface (Respiralogics, Reno, NV), a version of SBPs, replaced RAM as primary interface for BCPAP in October 2019. The RAM cannula remained available and was utilized for documented nasal irritation. Throughout study dates, we utilized a single outlet bubble device (B&B Bubbler, Carlsbad, CA) with a Flow rate of 8 L/min attached to an oxygenblender. Previous bench testing experiments demonstrated a pressure transmission of RAM cannula Green-Preemie sized has a 75.8% transmission of bottle pressure whereas the Babi.Plus size 1 or 2 has a 96% transmission, with a 23% leak at the nose for both interfaces [4, 9]. Respiratory therapists typically record the nasal interface type, size, and pressures in the EPIC flow sheet with each assessment.

Data was electronically extracted using Microsoft SQL, and programing code was confirmed with selective chart review [21]. SQL programming can extract specific flow sheet data on vital signs (i.e., FiO_2_, respiratory rate, oxygen saturations), respiratory device support (CPAP, Ventilator, or non-invasive mechanical ventilation (NIPPV)), type of nasal interface (RAM or Babi.Plus), CPAP pressures, apnea events, and laboratory values for specific time points. Vital signs and respiratory variables for each patient were averaged for every 2 h period of time. Time points of interest analyzed included: 1) Delivery room, 2) First 72 h of life in infants admitted to the NICU on BCPAP, 3) First 72 h after first initial extubation attempt if conducted within the first 14 days of life (infants requiring intubation), and 4) 31 0/7 weeks post-menstrual age for infants born less than 30 weeks PMA. The first extubation attempt within first 14 days of life time-point was determined electronically from flowsheets and confirmed by selective chart review. 31 0/7 week time point was based on the gestational age at birth. Infants were assigned to a BCPAP nasal interface based on type charted by respiratory therapist, and all infants prior to October 2019 were assigned to RAM cannula as both units switched interfaces on a specific day. Infants were resuscitated per NRP guidelines and PPV was provided with a T-piece resuscitator and not via either nasal interface. Use of PPV in the delivery room was electronically extracted from the Newborn Resuscitaiton form built into EPIC. Infants who were intubated in delivery room were assigned to the nasal interface and pressure most commonly used in the 3 months before and after the delivery. Infants extubated to NIPPV were assigned to group by the first BCPAP nasal interface and pressure after NIPPV, but were considered CPAP failures for analysis. CPAP failure was defined as intubation or non-invasive mechanical ventilation within 72 h of time point of interest. Clinical guidelines in our unit considered an infant to have failed bCPAP if they have >6 apnea events/hour or apnea requiring bag-mask ventilation for recovery, a need for oxygen greater than FiO_2_ 0.40 to maintain saturations >90%, or a PCO_2_ > 65 mmHg with a pH less than 7.20. Infants who failed CPAP received surfactant via the endotracheal tube, with no infants receiving minimally invasive surfactant during timeframe studied. Caffeine was typically started within the first few hours on infants less than 32 weeks. Reason for use of RAM cannula after October 2019 (Babi.Plus cohort) was determined from physician notes.

### Statistical analysis

Values reported as Mean ± Standard deviation, median (IQR), or percentage, dependent on the distribution of the variable. Continuous data were analyzed with independent samples = *t-*tests or Mann-Whitney tests when values were not uniformly distributed. One-way ANOVA with Tukey or Bonferroni correction for multiple comparisons were used and results are presented as Mean ± standard deviation (SD) (Graph Pad Prism 10.2). Chi-square tests or Fisher’s Exact tests were used as appropriate for categorical variables (Prism 10.2, Vassarstats.net). Statistical significance was set at *p* < 0.05.

## Results

There were 2432 infants less than 33 weeks gestational age delivered at St. Mary’s Hospital between January 2013 and December 2023. Babi.Plus (Babi) prongs (short-nasal prongs) were introduced in October 2019, and replaced the RAM cannula (Green-Premie) as the primary nasal interface for CPAP (Table 1). CPAP pressures of 5 and 6 cmH_2_O (5/6), and 7 and 8 cmH_2_O (7/8) were combined for analysis. Calculated transmissioned nasopharyngeal pressures for RAM5/6 was 4.38 ± 0.3 cmH_2_O, whereas RAM7/8 was 5.71 ± 0.5 cmH_2_O and nearly identical to the Babi5/6 at 5.74 ± 0.5 cmH_2_O. There were no differences in the birthweights, gestational ages, or the percent of infants with any amount of PPV received in delivery room between groups (Table 1). Less infants required intubation in the delivery room in both RAM7/8 (23%) and Babi5/6 (28%) than RAM5/6 (35%), and the RAM7/8 failure rate was slightly lower than Babi5/6 (*p* < 0.05) (Table 1). In infants born less than 29 weeks GA, less infants required intubation in the delivery room in the both RAM7/8 (49%) and Babi5/6 (56%) than RAM5/6 (65%) with no differences between Babi5/6 and RAM7/8 (Supplementary Table 1).

**Table 1:** Delivery room and NICU admission for infants less than 33 weeks born.

		RAM 5/6	RAM 7/8	BABI 5/6
**Delivery room**				
Number of Infants	n	1039	673	720
Gestational Age	weeks	29.3 ± 2.7	29.5 ± 2.7	29.5 ± 2.6
Birth Weight	grams	1258 ± 439	1300 ± 448	1339 ± 469*
5 min Apgar		8 (7-9)	8 (7-9)	8 (7-9)
Received PPV in DR	# (%)	602 (58%)	376 (59%)	412 (58%)
Intubation in DR	# (%)	365 (35%)	152 (24%)*	201 (28%)*
**First 72 h of infants admitted to NICU on CPAP**				
Number of infants	n	636	379	372
Gestational age	weeks	30.5 ± 1.9	30.2 ± 2.1	30.1 ± 2.0
Birth weight	grams	1387 ± 373	1376 ± 422	1413 ± 426
CPAP pressure at bottle	cmH_2_O	5.8 ± 0.4	7.5 ± 0.6*	6.0 ± 0.5
Adjusted CPAP for Interface	cmH_2_O	4.38 ± 0.3	5.71 ± 0.5*	5.74 ± 0.5*
pCO_2_ in first 2 h	mmHg	57 ± 13	58 ± 13	54 ± 13*#
pCO_2_ > 65 mmHg in 72 h	# (%)	104 (16%)	68 (18%)	54 (15%)
FiO_2_ > 40% in 72 h	# (%)	126 (20%)#	116 (31%)	90 (24%)
Apnea events >6 per 24 h	# (%)	37 (5.8%)	13 (3.4%)	11 (3.0%)*
Mechanical ventilation in 72 h	# (%)	157 (25%)	109 (29%)	77 (21%)*#
Intubation in DR or NICU	# (%)	522 (50%)	261 (39%)*	278 (39%)*

Mean ± SD; Median (IQR 25-75) * *p* < 0.05 vs RAM 5-6; # *p* < 0.05 vs RAM 7-8.

### Infants admitted to NICU on CPAP

Infants admitted to the NICU on CPAP had similar birth weight, gestational ages, and Apgar scores between the groups (Table 1). Maximum PCO_2_ in the first 2 h was lowest in the Babi5/6 group at 54 ± 13 mmHg compared to RAM5/6 57 ± 13 and RAM7/8 58 ± 13 (*p* < 0.05). There were no differences in the percent of infants with PCO_2_ > 65 mmHg in first 72 h. Babi5/6 had less infants with > 6 apnea events in 24 hs (3.0%) than RAM5/6 (5.8%) (*p* < 0.05) but similar to RAM7/8 (3.4%). The average FiO_2_ for first 12 h was lower in RAM5/6 (27%) and Babi5/6 (28%) than the RAM7/8 (30%) (*p* < 0.0001). The failure rate of CPAP in the NICU was lowest in the Babi5/6 group (21%) compared to RAM5/6 (25%) and RAM7/8 (29%) (*p* < 0.05 vs both groups). When CPAP failure in DR is added to NICU failure, the RAM7/8 (39%) and Babi5/6 (39%) had identical rates, which were lower than the RAM5/6 (50%) (*p* < 0.05). In infants <29 weeks, the combined rates of failure in DR or NICU were similar between RAM7/8 (69%) and Babi5/6 (71%) and lower than RAM5/6 (79%) (*p* < 0.05, Supplementary Table 1). There were no differences in pneumothorax rates between groups.

### Extubated within the first 14 days of life

Infants who received intubation but were extubated within the first 14 days were evaluated for first 72 h after extubation. The initial CPAP pressure used or whether non-invasive positive pressure ventilation (NIPPV) was determined by the clinician and would likely have been influenced by the severity of illness or gestational age. Some infants were extubated to Babi.Plus 7 or 8 cmH_2_O. Infants extubated to RAM7/8 or Babi7/8 had lower gestational ages and birth weights than RAM5/6 or Babi5/6, and slightly lower oxygen requirements (27% vs 30%) in 4 h prior to extubation (Table 2). Infants in the RAM cohort were less likely to be extubated to NIPPV (218/945 infants, 23%) than Babi.Plus Cohorts (104/364 infants, 29%) (*p* = 0.04). The infants in Babi.Plus cohort to NIPPV were statistically similar sized (871 ± 300 grams vs 926 ± 342 grams, *p* = 0.16) and gestational age at birth (26.5 ± 2.7 GA vs 27.1 ± 2.4, *p* = 0.052), though trended to be smaller. The differences were driven by infants extubated to NIPPV then placed on Babi 7/8, who were smaller and earlier (Supplementary Table 2). When infants extubate to CPAP and NIPPV were combined, there were no differences between BW (RAM 1160 g vs BABI 1180 g, *p* = 0.28) or gestational age (RAM 28.4 vs Babi 28.4 weeks, *p* = 0.98) between the cohorts.

**Table 2:** Respiratory outcomes in first 72 h after first extubation to CPAP.

		RAM 5/6	RAM 7/8	BABI 5/6	BABI 7/8
**Pre-extubation Information**					
Number of Infants	n	419	310	188	72
Gestational Age	weeks	29.1 ± 2.5	28.4 ± 2.6*	29.3 ± 2.4#	28.8 ± 2.2
Birth Weight	grams	1285 ± 439	1154 ± 440*	1358 ± 447#	1209 ± 460
DOL Extubation	day	3.3 ± 4.8	5.4 ± 8.2	3.3 ± 6.6	4.7 ± 6.9
FiO_2_ 4 h prior to extubation	% O_2_	27 ± 8	30 ± 9*&	27 ± 9#	30 ± 9*&
**Within the 72 h after extubation**					
Change FiO_2_ from Vent 4 h	% O_2_	3.1 ± 7.8	4.0 ± 7.6	2.7 ± 5.2	2.5 ± 8.1
Infants with >15% increase	# (%)	28 (6.8%)	17 (5.5%)	7 (3.7%)	2 (2.7%)
FiO_2_ first 24 h	% O_2_	29 ± 11	32 ± 10*&	28 ± 10#	30 ± 9
FiO_2_/saturation ratio		0.31 ± 0.12	0.34 ± 0.10*	0.30 ± 0.11#	0.32 ± 0.10#
Maximum CO_2_ value in 24 h	mmHg	44 ± 10	45 ± 10	42 ± 10#	45 ± 9
Infants with CO_2_ > 65 mmHg	# (%)	10 (2.3%)	7 (2.3%)	6 (3.2%)	1 (1.4%)
Increased CPAP pressure	# (%)	127 (30%)	67 (22%)*	63 (34%)#	14 (19%)
Apnea >6 in 24 h	# (%)	50 (13%)	39 (12%)	8 (4%)*#	6 (8%)
CPAP Failure in 72 h	# (%)	109 (26%)	76 (25%)	29 (15%)*#	7 (10%)*#

Mean ± SD. * *p* < 0.05 vs RAM 5/6; #*p* < 0.05 vs RAM 7/8; & *p* < 0.05 vs Babi 5/6.

Of infants extubated to BCPAP (Table 2), there were no differences in the increase in oxygen requirement between pressures or nasal interface after extubation. Subtle differences in oxygen and ratio of oxygen to saturations between groups that existed prior to extubation persisted babies after extubation. There were no differences in the percent of infants with hypercarbia between groups, but a higher percent of infants in the RAM5/6 (30%) and Babi5/6 (34%) requiring increased pressures from initial pressure after extubation (Table 2). Babi5/6 had the percent of infants with Apnea >6 per 24 h (4%) compared with RAM5/6 (13%) and RAM7/8 (12%) (*p* < 0.05 vs both groups). Fewer infants in the Babi5/6 group failed CPAP after extubation (15%) than in the RAM5/6 (26%) and RAM 7/8 (25%) (*p* < 0.05 for both groups). If we assume any infant extubated to NIPPV would have failed CPAP, adding these infants into analysis, we find Babi5/6 continues to have lower failure rate (31%) compared with RAM5/6 (44%) or RAM7/8 (41%) (*p* < 0.05 vs both groups).

### At 31 weeks post-menstrual age

At 31 weeks PMA, 29% of infants initially started on Babi.Plus prongs were on the RAM cannula. Of infants switched to RAM cannula from Babi.Plus, 48% of infants had nasal irritation or ulceration as the reason for changing nasal interfaces documented in the physician note, and another 35% of these infants were extubated to NIPPV via the Ram cannula and remained on RAM when converted to BCPAP. Infants at 31 weeks were analyzed based on the CPAP interface they were currently on and the pressure currently being used. Infants in the RAM5/6 and Babi 5/6 were slightly more mature at birth than infants on RAM7/8 or Babi7/8 (Table 3). Oxygen requirements were higher in the infants on RAM7/8 or Babi7/8 for similar saturations and respiratory rates. Maximum PCO_2_ was similar between groups. Infant on Babi5/6 had lower average number of apneas per 24 hours and lower present of infants with apnea.

**Table 3:** Respiratory outcomes at 31 0/7 weeks PMA in infants on CPAP.

		RAM 5/6	RAM 7/8	BABI 5/6	BABI 7/8
**Pre-extubation Information**					
Number of Infants	n	444	366	195	80
Gestational Age	weeks	27.6 ± 1.7	26.8 ± 1.8*&	27.8 ± 1.7	26.1 ± 1.8*#&
Birth weight	grams	1015 ± 276	919 ± 264	1070 ± 280	848 ± 232*&
Average FiO2	%	26 ± 7	30 ± 9*&	25 ± 9	32 ± 12*&
Average Saturations	%	96 ± 3	95 ± 3*	96 ± 3	95 ± 2&
Average Resp Rate	bpm	53 ± 8	55 ± 9*	52 ± 10#	53 ± 8
Average Apnea events/24 h	#	0.93 ± 2.2	1.0 ± 1.7	0.32 ± 0.79*#	0.87 ± 1.7&
Infants with Apnea Events	#(%)	222 (50%)	192 (52%)	61 (31%)*#	45 (56%)&
Max CO_2_ in 72 hours	mmHg	53 ± 10	52 ± 8	53 ± 10	53 ± 9

Mean ± SD. * *p* < 0.05 vs RAM 5/6; #*p* < 0.05 vs RAM 7/8; & *p* < 0.05 vs Babi 5/6.

## Discussion

**Fig. 1:**
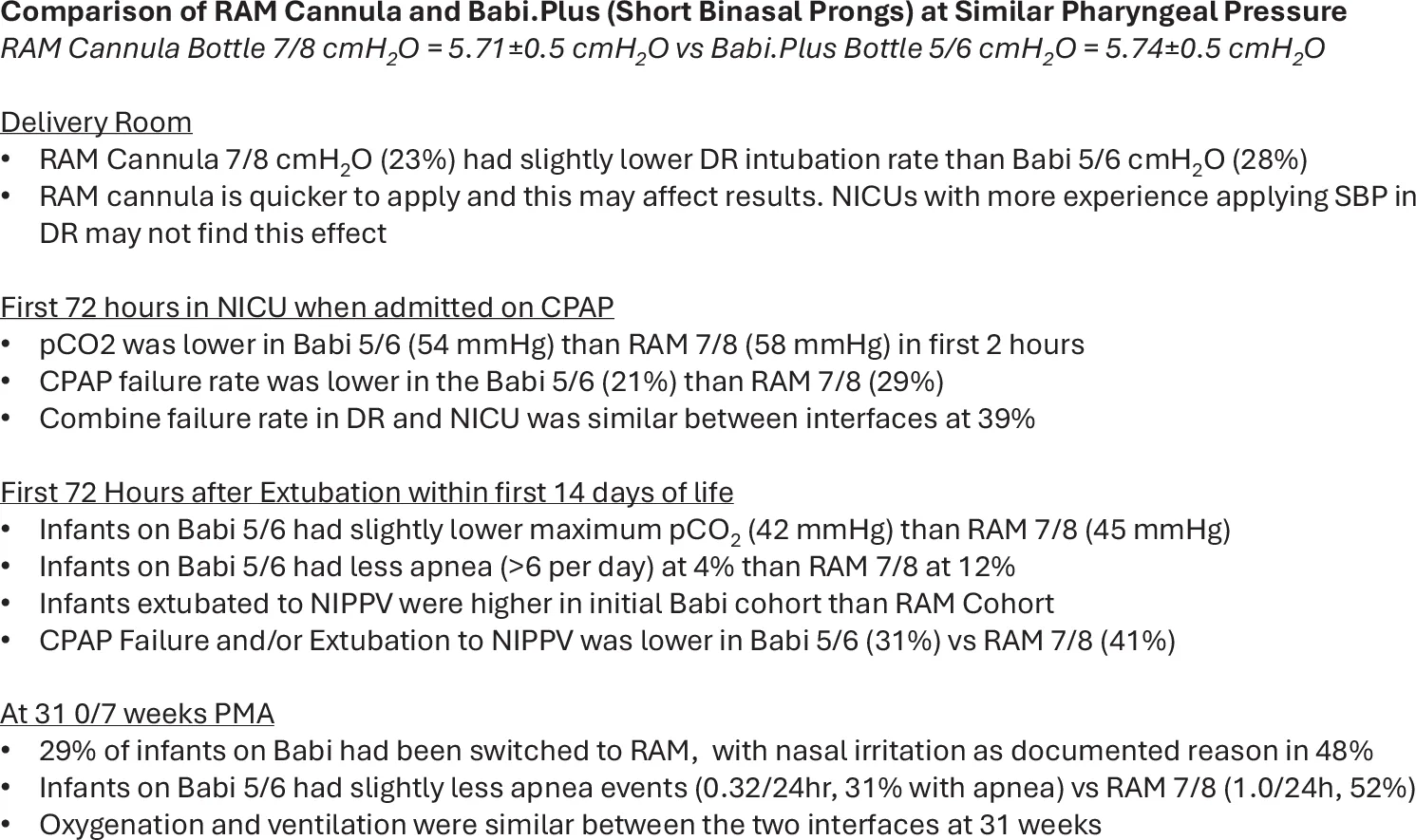
Comparison of RAM Cannula and Babi.Plus (Short Binasal Prongs) at Similar Pharyngeal Pressure.

In this retrospective review of the hospital course of over 2400 infants born less than 33 weeks gestational age, we demonstrated a small but significant advantage of the Babi.Plus (SBPs) on CPAP failure and apnea over RAM cannula. Even when bottle pressures were adjusted to equal nasopharyngeal pressures [RAM 7/8 cmH_2_O (bottle) = 5.71 cmH_2_O (nose) vs Babi 5/6 cmH_2_O (bottle) = 5.74 cmH_2_O (nose)], the differences persist. We again demonstrated the benefit of higher CPAP bottle pressures with the RAM CPAP ( > 7 cmH_2_O) throughout the hospitalization compared to RAM pressures of 5 or 6 cmH_2_O. There are benefits of RAM cannula at higher pressures in the delivery room, so failure rates in either DR or NICU in the first 72 h were similar when distending pressure was similar (RAM 7/8 cmH_2_O or Babi.Plus 5/6 cmH_2_O) (Fig. 1). There were no advantages in using 5 or 6 cmH_2_O with RAM cannula at any stage in the hospitalization. These results were similar when examining both all infants less than 33 weeks gestational age and those less than 29 weeks gestational age, though more infants in lower GA group fail CPAP overall. There were a significant number of infants (29%) of infants on the Babi.Plus interface that were switched to the RAM by 31 weeks and nasal breakdown was reason noted for change in the majority.

Applications of BCPAP nasal interface in the delivery room is a challenging task at a time sensitive moment of transition to air breathing. The RAM cannula may be more easily applied in the delivery room thus achieving CPAP quicker than with the Babi.Plus system. Consistent and rapid recruitment of FRC likely improves the transition at birth and the clearance of lung fluid [22] The pressure of the CPAP mask on the infant during resuscitation can increase bradycardia and apnea events, especially in less experienced resuscitators, so a more rapid transition with RAM cannula 7/8 cmH_2_O could decrease the resuscitator’s desire to intubate the preterm infant [23] Rapid placement of the RAM cannula in the delivery room may explain differences in the DR intubation rates between RAM 7/8 and Babi 5/6. Binasal prongs, in simulations, provided more constant CPAP than mask CPAP and thus more quickly establishing the BCPAP may have a benefit [24] The Babi.Plus interface is slightly more bulky and takes longer to place in our experience. Units that have been using BCPAP with Hudson prong in the delivery for a long time are very efficient at getting them attached, and may not find a time advantage with RAM. Introduction of early CPAP with SBPs in the delivery room decreased the rates of severe BPD at Montreal Children’s, and rates improved as they became more comfortable with the process [14] With the increased use in less invasive surfactant administration in NICUs, stabilization with CPAP remains crucial as need for intubation following LISA has been associated with a higher rate of death or BPD [25] Quality improvement projects directed at rapid, early application of BCPAP may be helpful in understanding these effects [12].

Decreased apnea events seen in our infants with the Babi.Plus prongs may be due to activation of stretch receptors (both inflation and deflation types) in the lungs or may work through irritant receptors in the airways [26] Mechanosensitive Piezo channels are present in the developing lung within the fetal airway smooth muscle and are important for airway development [27] The increased transmission of the bubble pressure fluctuations may provide additional respiratory drive benefits. Compared with ventilator derived CPAP, BCPAP increased oxygenation and ventilation found in preterm lambs through recruitment of more of the lung when measured by sulfurhexafluoride washout [10] Increasing the intensity and variation of pressure oscillations by elongating/angulating the bubble outflow arm (Seatle-CPAP) created improved oxygenation of juvenile rabbits compared with standard CPAP [11] Unfortunately, when infants were randomized to Seatle-CPAP or BCPAP with the Fisher-Paykel system, there were no differences in the rates of CPAP failure or other respiratory outcomes [28] This may partially due to the highly variable pressure of the Fisher-Paykel bottle when compared to other available bottles [17] Across Canadian NICUs, extubation to CPAP was not inferior to NIPPV, and the failure rates were similar to our rates of around 30% [29] Even when we assigned all infants who were extubated to NIPPV as failed CPAP, the Babi5/6 group had lower extubation failure than either of the RAM cannula groups.

A large portion (29%) of the infants on the Babi.Plus interface were changed to a RAM cannula by 31 weeks post-menstrual age and most of these were charted as due to nasal irritation. The Babi.Plus system has very soft nasal prongs but a stiff plastic tubing that extends beyond the face, and efforts to keep prongs in place may lead to nasal trauma. Nasal trauma from CPAP prongs is one of the most common reasons clinicians move away from CPAP, but this may be due to improper positioning of the prongs [30] A QI project at George Washington University Hospital found nasal injury was associated with not following the nursing checklist, excessive water condensation in the CPAP tubing, and incorrect placement of the binasal prong bridge [30] Nasal injury from prongs is very common, in over 40% in all infants in a recent study, and rotation between mask and nasal prongs may improve nasal injury [31]. Mask BCPAP has less nasal injury than nasal prongs, though rotating the devices did not provide additional benefits for nasal injury prevention in this systemic review [32]. Prophylactic use of hydrocolloid dressings around the nostrils may provide some protection against nasal injury [32]. Our unit uses non-sting barrier and close monitoring of nasal septum with rotation of interfaces when needed. In randomized study of prolonged CPAP, no infants required discontinuing CPAP for nasal injury or feeding intolerance suggesting Hudson prongs or mask CPAP may not have same nasal irritation seen with the Babi.Plus in our unit [5]. In an attempt to maintain the benefits of the short nasal prongs on pressure transmission and apnea events, we have recently introduced a third BCPAP interface (Fisher and Paykel Flexi-trunk and Mask) to reduce the nasal irritation. It is too early in the process to determine its efficacy.

There continues to be a debate between Neonatologists about whether the RAM cannula is closer to the humidified high flow nasal cannula (HHFNC) than to BCPAP. Unlike other nasal interfaces used for BCPAP, including the Babi.Plus, the RAM cannula does not have an exhalation limb, the RAM cannula is not designed to occlude the nares, and CPAP is generated in the cap of the cannula versus in the nasopharyngeal space. In this study, we demonstrated differences in clinical outcomes between a RAM CPAP pressure of 5/6 cmH_2_O and 7/8 cmH_2_O, while maintaining the same gas flow rates and cannula sizes. This difference in clinical outcomes based on pressure, along with our previous bench testing and that by Fernando et al., would suggest CPAP pressure is created in the nasal pharynx more than high flow alone [4, 20, 33] The pressure fluctuations measured created by the bubbling are still present, though diminished, with RAM cannula attached to the BCPAP bottle, and bubbling can be heard throughout the lungs in infants. The pressure difference could be due to transmission of variable pressures for the cap of the RAM cannula, or through changes in air flow rates as pressure overcomes resistance in the tubing. If someone considers RAM cannula as HHFNC, it is not surprising that it was not as successful based on previous randomized trials (HUNTER, HIPSTER) showing a small benefit, though non-inferior, of CPAP over HHFNC [34] The RAM cannula (RAM CPAP) likely functions as a hybrid between BCPAP and HHFNC, giving the benefits of HHFNC (less injury) but the disadvantage of more CPAP failure, especially at low set bottle pressures.

There are several limitations due to the retrospective nature of the analysis and conclusions that include two assumption designed to reduce inadvertent biases. The first assumption was that infants in the delivery room were assigned a CPAP pressure based on the average CPAP pressure used upon arrival in the NICU during the 6 months surrounding their birth. This was done because selective chart review of over 20% of infants could not determine the exact CPAP pressure used in delivery room nor the exact timing of intubation in the delivery room. This assumption about DR pressures should be minimized by combining two pressures (RAM5/6, RAM7/8, and Babi5/6) and allowed inclusion of this time point in the analysis. Not including the need for intubations in the DR for RAM5/6 would have skewed the data on 72 hours mechanical ventilation since this group had a much higher percentage fail. The speculation that time to apply RAM cannula in delivery room is anecdotal, as no one timed these occurrences. The second assumption was to assign all infants extubated to NIPPV to the CPAP failure group when some of these infants may have done well on BCPAP. Since there were differences between the infants placed on NIPPV between groups, not including these infants would have artificially skewed the infants in one direction. Even with assuming all the babies failed CPAP, there was a benefit of Babi.Plus on CPAP failure, strengthening the claim for a difference. These infants were assigned to the first CPAP level noted after NIPPV, and it may be that sicker, younger infants stayed on the NIPPV longer and level eventual CPAP level was lower. The CPAP level chosen at extubation may have been due to the clinical scenarios as infants extubated to higher pressures were slightly smaller, earlier gestational ages, and had higher oxygen requirements at time of extubation.

The differences in CPAP failure rates and ventilation differences are small, though significant, and may have less clinical relevance to justify introduction of a new interface. A combined approach to nasal interfaces, similar to our BCPAP protocol for transport which maximizes the RAM pressures prior to changing to SBP, may be appropriate in some settings. Infants with increased risk of apnea or for whom mildly better oxygenation would prevent intubation might benefit from SBPs whereas the average infant with mild RDS might do just as well on RAM with higher pressures [35].

## Conclusions

In summary, SBPs, specifically Babi.Plus, in our ten year experience were associated with slightly less CPAP failure in the first 72 h in NICU and after extubation, likely due to a decrease in apnea events. RAM cannula, when pressure was increased to match SNP pressures, had slightly less CPAP failure in the DR than Babi.Plus and similar results by 31 weeks CGA. RAM CPAP at 5/6 cmH_2_O had increased CPAP failure rates throughout hospitalization and should be avoided due to inadequate distending pressures. Early application of CPAP in the DR is critical for assisting with airway fluid clearance and development of respiratory drive. Institutions with a lot of experience with SNP may be able to achieve application as quickly as the RAM cannula, and may not see differences observed in our study. The differences in performance are small once pressure transmission is corrected, so institutions should consider all variables (cost, clinician comfort, nasal irritation, current CPAP failure rates, etc.) when deciding on a nasal interface for BCPAP. In an ideal situation where adequate respiratory therapy and/or nursing is available to decrease nasal irritation and maintain good nasal seal, the early application of short nasal prongs in the delivery room and throughout the NICU may decrease the need for mechanical ventilation and apnea events.

## Supplementary information

Supplemental Tables

## Data Availability

Primary data is available upon request from the corresponding author.
