# Supplementary material for: Comparison of nasal interfaces and pressures during critical time-points in NICU: observation data from over 10 years
Source: J Perinatol. 2026 Mar 30;46(10):2016–22. doi: 10.1038/s41372-026-02642-9 (PMC13680264; doi:10.1038/s41372-026-02642-9)
Supplement: Supplementary file 1 [file 41372_2026_2642_MOESM1_ESM.docx]

| **Supplemental Table 1: Delivery room and NICU admission for infants less than 29 weeks born** | | | | |
| --- | --- | --- | --- | --- |
|  |  | **RAM 5/6** | **RAM 7/8** | **BABI 5/6** |
| **Delivery Room** | | | | |
| Number of Infants | n | 408 | 237 | 262 |
| Gestational Age | weeks | 26.5±1.6 | 26.6±1.7 | 26.5±1.6 |
| Birth Weight | grams | 868±235 | 884±232 | 902±256 |
| 5 Min Apgar |  | 8 (7-8) | 8 (7-8) | 8 (7-9) |
| Intubation in DR | # (%) | 269 (65%) | 116 (49%)* | 146 (54%)* |
| **First 72 hours of infants admitted to NICU on CPAP** | | | | |
| Number of Infants | n | 132 | 109 | 92 |
| Gestational Age | weeks | 27.6±1.1 | 27.4±1.2 | 27.3±1.0 |
| Birth Weight | grams | 996±221 | 948±238 | 979±257 |
| pCO_2_ in first 2 Hour | mmHg | 55±10 | 53±10 | 52±11 |
| pCO_2_ >65 mmHg in 72 h | # (%) | 17 (13%) | 10 (9%) | 11 (12%) |
| FiO_2_ > 40% in 72 h | # (%) | 44 (33%) | 53 (48%)* | 38(42%) |
| Apnea Events >6 per 24h | # (%) | 13 (10%) | 9 (8%) | 5 (5%) |
| Mechanical Ventilation in 72 hours | # (%) | 55 (42%) | 47 (43%) | 40 (43%) |
| MV in DR or NICU | # (%) | 324 (79%) | 163 (69%)* | 186 (71%)* |
| Mean±SD, Median (IQR 25-75); * p<0.05 vs RAM 5-6; # p<0.05 vs RAM 7-8 | | | | |

| **Supplemental Table 2: Infants extubated to NIPPV assigned by first CPAP level after NIPPV** | | | | | |
| --- | --- | --- | --- | --- | --- |
|  |  | **RAM 5/6** | **RAM 7/8** | **BABI 5/6** | **BABI 7/8** |
| Extubated Infants | n | 549 | 396 | 232 | 132 |
| Number to CPAP | n | 419 | 310 | 188 | 72 |
| Number to NIPPV | n | 130 | 86 | 44 | 60 |
| % to NIPPV | % | 24% | 22% | 20% | 45%* |
| Gestational Age | weeks | 27.3±2.5 | 26.7±2.4 | 27.2±2.4 | 26.0±1.9* |
| Birth weight | grams | 944±323 | 898±369 | 965±376 | 803±207* |
| CPAP Failure + NIPPV | N (%) | 239 (44%) | 162 (41%) | 73 (31%)*# | 67 (52%)& |
| Mean±SD. * p<0.05 vs RAM 5/6; #p<0.05 vs RAM 7/8; & p<0.05 vs Babi 5/6 | | | | | |
